# The Cost of Providing District-Level Surgery in Malawi

**DOI:** 10.1007/s00268-017-4166-5

**Published:** 2017-08-08

**Authors:** Dennis Cornelissen, Gerald Mwapasa, Jakub Gajewski, Tracey McCauley, Eric Borgstein, Ruairi Brugha, Leon Bijlmakers

**Affiliations:** 1 Radboud University Medical Centre Netherlands, Geert Grooteplein Zuid 10, 6525 GA Nijmegen, The Netherlands; 2 College of Medicine, University of Malawi, Mahatma Gandhi, Blantyre, Malawi; 3 Royal College of Surgeons in Ireland, 123 St Stephens Green, Dublin 2, Ireland

## Abstract

**Background:**

Three district hospitals in Malawi that provide essential surgery, which for many patients can be lifesaving or prevent disability, formed the setting of this costing study.

**Methods:**

All resources used at district hospitals for the delivery of surgery were identified and quantified. The hospital departments were divided into three categories of cost centres—the final cost centre, intermediate and ancillary cost centres. All costs of human resources, buildings, equipment, medical and non-medical supplies and utilities were quantified and allocated to surgery through step-down accounting.

**Results:**

The total cost of surgery, including post-operative care, ranged from US$ 329,000 per year to more than twice that amount at one of the hospitals. At two hospitals, it represented 16–17% of the total cost of running the hospital. The main cost drivers of surgery were transport and inpatient services, including catering. The cost of a C-section ranged from $ 164 to 638 that of a hernia repair from $ 137 to 598. Evacuations from uterus were cheapest mainly because of the shorter duration of patient stay.

**Conclusion:**

Low bed occupancy rates and utilisation rates of the operating theatres suggest overcapacity but may also indicate a potential to scale up surgery. This may be achieved by adding surgical staff, although there may be rate-limiting steps, such as demand for surgery in the community or capacity to provide anaesthesia. If a scale-up of surgery cannot be realised, hospital managers may be forced to reduce the number of beds, reorganise wards and/or reallocate staff to achieve better economies of scale.

**Electronic supplementary material:**

The online version of this article (doi:10.1007/s00268-017-4166-5) contains supplementary material, which is available to authorized users.

## Introduction

Surgery has for long been neglected in low- and middle-income countries (LMIC), as a result of which more than 95% of the population in South Asia and most of sub-Saharan Africa do not have access to basic surgical care [1]. In 2010, an estimated 16.9 million lives were lost from conditions needing surgical care; this represented almost a third (32.9%) of all deaths worldwide [2]. Only 6% of the 313 million procedures undertaken each year occur in the poorest countries, where over a third of the world’s population lives, and 143 million additional surgical procedures are needed each year to save lives and prevent disability [3].

One of the four working groups of the Lancet Commission on Global Surgery focused on the economics and financing of surgical and anaesthesia care. The group has shown that without an accelerated investment in the scale-up of surgery, LMIC will face enormous losses in economic productivity which may accumulate to around US$ 12 trillion between 2015 and 2030 [3]. The annual value of lost economic output secondary to surgical conditions will have a profound effect, especially in LMIC, where by the year 2030 surgical conditions could reduce annual growth in gross domestic products by almost 2%.

While the need for functional health systems and the structural availability of human resources, infrastructure and supplies to deliver essential surgery is clear, financing is uncertain in many LMIC and financial mechanisms inadequate. Moreover, the way health services are organised and financed in most countries does not allow a good insight into how much it actually costs to provide essential surgery, leave alone how much extra money would be required to scale up surgery in order to meet the demand.

Malawi’s national health care system is organised around district hospitals which provide first-level services to patients who are referred by primary health care units. These district hospitals in turn refer certain patients to one of the four central hospitals which provide specialist care. The bulk of the clinical care at district hospitals is provided by clinical officers. These are non-physician clinicians who, amongst many other tasks, undertake obstetric, gynaecological and basic general surgery, of which the post-operative outcomes are comparable to procedures that are performed by medical officers [4, 5]. Since 2013, the University of Malawi College of Medicine has been providing training in surgery to clinical officers, through a BSc in surgery, that received funding during the first four years through the Clinical Officers Surgical Training in Africa project (COST-Africa, see www.costafrica.eu). This matched pair randomised controlled trial (RCT) research project, which was funded by the European Union under its Framework Programme 7, aimed to demonstrate the effectiveness and cost-effectiveness of increasing the surgical capacity of non-physician clinicians in rural Malawi and Zambia. The trial was registered in the ISRCTN registry on 27 February 2014, under registration number 66099597.

This paper identifies the resources and estimates the costs of delivering surgery at district hospitals in Malawi; it expresses the cost of surgery as a percentage of the total annual cost of running a district hospital and calculates unit costs of selected surgical procedures.

In order to determine the annual costs of providing surgery, two questions guided the study:

1. What is the cost of surgery, taking into account not just resources that are used in the operating theatre (OT) and on the surgical wards, but also inputs such as staff time and transport facilities, that are shared with other hospital departments for services other than surgery?
2. What are the unit costs of common surgical procedures conducted at district hospitals in Malawi?

Three district hospitals formed the setting of the study. Each had received two COST-Africa sponsored BSc students, who were undergoing in-service training through weekly visits from surgeon trainers. The hospitals were selected for this economic analysis out of eight candidates, mainly for reasons of convenience: Mangochi district hospital (MaDH), Mulanje district hospital (MuDH) and Nsanje district hospital (NsDH), all situated in the country’s southern region. The three hospitals are similar in terms of infrastructure and service packages offered. Each has one main operating theatre for major surgery cases and a smaller theatre for minor cases; in addition, MuDH and NsDH each have a small operating theatre within the outpatient department (OPD) for minor surgery. None of the three hospitals have designated surgical wards.

## Materials and methods

OT registers were used to count the number of surgical procedures performed over a 12-month period (from 1 July 2013 to 30 June 2014) and categorise them into four types: pregnancy-related and obstetric surgery, emergency and disability preventive surgery, injury-related surgery and other major or minor procedures, as shown in Box 1.

**Box 1:** Types of surgical procedures by category

Pregnancy-related and obstetric surgery	Emergency and disability-preventive surgery	Injury-related surgery	Other surgery
Caesarean section Hysterectomy Repair of ruptured uterus Salpingectomy Elective bilateral tubal ligation (BTL) BTL in combination with a caesarean section Dilatation and curettage/manual vacuum aspiration Evacuation of uterus Other major gynaecological procedures Other minor gynaecological procedures	Inguinal herniotomy/herniorrhaphy Femoral hernia repair Other hernia’s (umbilical, epigastric, incisional) Hydrocoele repair Correction of torsion Prostatectomy Appendectomy Gastro-intestinal resection and anastomosis Explorative/other laparotomy Amputation/disarticulation Cataract removal Circumcision	Manipulation under anaesthesia Suturing Debridement Skin grafting, dressing	Other major procedures, such as Vesico-vaginal fistula repair Myomectomy Decompression Minor procedures Foreign body removal Incision and drainage Excision

Step-down accounting was applied for costing. This is a relatively simple method that is recommended by the World Health Organization for generating costs and unit cost data at the health facility level in low- and middle-income countries. It identifies and quantifies the various inputs used by a health facility, estimates the total cost of each of these inputs, allocates them to cost centres and links them to the various services that are provided [6, 7]. It involves six steps of which detailed descriptions are given in Online Resource 1: (1) define the final product(s) of interest; (2) define the final, intermediate and ancillary cost centres; (3) identify all inputs and calculate the full costs of each input; (4) assign inputs to the appropriate cost centres; (5) allocate all direct costs and appropriate proportions of the costs of the inputs used at ancillary and intermediate cost centres to the final cost centre; and (6) calculate the unit cost for each service provided at the final cost centre.

Data collection took place between November 2014 and March 2015, and all data pertain to the financial year July 2013–June 2014. Online Resource 1 also describes the electronic model that was constructed to enter the data into spreadsheets, allocate costs to surgery, calculate unit costs of individual surgical procedures and estimate the cost implications of various alternatives for scaling up district-level surgery.

## Results

The key parameters of the three hospitals involved in the study are shown in Table 1.

**Table 1:** Key parameters of Mangochi, Mulanje and Nsanje district hospitals in 2013/14

	Ma DH	Mu DH	Ns DH
*Infrastructure*			
Surface area (in m^2^)	5439	4617	5364
Number of theatres inside the OT complex	2	2	2
Number of theatres located at the OPD	0	1	1
Hospital beds	263	244	228
Bed occupancy rate^a^	58%	50%	17%
Number of wards	6	5	6
Number of functional ambulances	12	9	9
Shortest route to the nearest central hospital^b,c^	127 km	61 km	173 km
*Staffing (excluding trainees/students and expatriate staff)*			
Medical officers	5	2	1
Clinical officers excluding COST-Africa sponsored CO’s	22	19	15
COST-Africa CO’s	2	3	2
Anaesthetists	1	3	3
Nursing officers	7	16	10
Laboratory and X-ray staff	7	8	11
Other (para-)medical staff	84	75	39
Administrative staff	29	27	28
Other support staff	221	214	193
TOTAL members of staff^d^	378	367	302
*Funding (in US$)*			
Government ORT budget^e^	920,407	800,301	375,986
External funding	239,431	169,800	347,262

^a^Bed occupancy rate (BOR) = number of beds times 365 divided by the number of inpatient days over a whole year

^b^All the three hospitals refer patients who need specialist treatment to Queen Elisabeth central hospital in Blantyre. MaDH also refers patients to Zomba central hospital, which is nearer but doesn’t have the full range of expertise and facilities of a typical referral hospital

^c^Calculated with Google Maps

^d^Excluding trainees/students who do not receive salaries

^e^Other recurrent transactions, from the annual budget allocation that hospitals and districts receive to cover the cost of various types of recurrent expenditure

Of note is the low bed occupancy rate at NsDH (17% compared to 50–58% at the other two hospitals), indicating overcapacity or under-utilisation of inpatient services. The total staff complement in each hospital ranged from 302 (at NsDH) to 378 (MaDH) of which about two-thirds were support staff such as hospital attendants, kitchen staff, mortuary attendants, grounds men and security guards. Clinical officers were the largest cadre of clinicians at these district hospitals, ranging from 17 to 24 per hospital, of which two (in MaDH and NsDH) or three (in MuDH) received additional surgical training through COST-Africa. MaDH had five medical officers, compared with two at MuDH and just one at NsDH. MaDH had only one trained anaesthetist versus three at the other two hospitals. In spite of its higher bed occupancy rate, MaDH had the fewest qualified nursing officers. MaDH and MuDH had much higher annual ORT budgets^1^ to cover recurrent expenditure than NsDH, but the latter hospital compensated this to some extent with more external funding (from donors), which brought in almost as much as the Government ORT funding.

### Surgical services and other hospital output

Table 2 shows the volume of patient services provided in the 2013/14 financial year per hospital, in terms of admissions and inpatient days for the main categories of surgical procedures.

**Table 2:** Surgical and non-surgical services provided by hospitals over 12 months (2013/14 fiscal year)

	Ma DH		Mu DH		Ns DH	
	Surgical procedures	Inpatient days	Surgical procedures	Inpatient days	Surgical procedures	Inpatient days
*Surgery-related patient services*						
Pregnancy-related and obstetric surgery	2060 (84%)	3048 (64%)	1526 (80%)	2351 (70%)	748 (53%)	3447 (61%)
Emergency and disability preventive surgery	162 (7%)	725 (15%)	141 (7%)	444 (13%)	243 (17%)	1254 (22%)
Injury-related surgery	97 (4%)	854 (18%)	113 (6%)	269 (8%)	289 (20%)	841 (15%)
Other surgical procedures	143 (6%)	120 (3%)	133 (7%)	276 (8%)	134 (9%)	116 (2%)
Total	2462 (100%)	4737 (100%)	1913 (100%)	3340 (100%)	1414 (100%)	5649 (100%)

	Admissions	Inpatient days	Admissions	Inpatient days	Admissions	Inpatient days
*Non-surgery-related inpatient services*						
Non-surgical inpatients	14,582	51,738	15,234	40,767	5077	10,005
*Outpatient services*						
OPD visits	161,192	n/a	109,828	n/a	87,610	n/a

MaDH conducted the largest number of surgical procedures (2462 cases), of which 84% involved pregnancy-related and obstetric surgery. MuDH came second (with just over 1900 cases of surgery), while NsDH had the smallest number (just over 1400 cases). The latter hospital had relatively more injury-related surgery (20% of all cases, compared with 4–6% at the other two hospitals) and more emergency and disability-preventive surgery (17% of all cases, compared with 7% at both MaDH and MuDH), which suggests that the demand for surgery at NsDH is less predictable. Just over half (53%) of all surgical cases at NsDH were for pregnancy-related and obstetric surgery, compared with 80% at MuDH and 84% of major surgical cases at MaDH. The workload in terms of inpatient days also differed between the three hospitals. While NsDH had the smallest number of surgical admissions, it had the largest number of surgical inpatient days.

In terms of patient load other than for surgery, MaDH was two to three times as busy as NsDH, having almost twice as many OPD visits, almost thrice as many admissions of patients other than for surgery, and five times as many non-surgical inpatient days.

### Costs

The total costs of providing surgery in each of the three hospitals over the 12-months period are presented in Table 3, with a breakdown by cost centre and type of expenditure.

**Table 3:** Cost of surgery (in US$) by cost centre and type of expenditure over 12 months (July 2013–June 2014)

	Ma DH	Mu DH	Ns DH
*Direct cost of operating theatre (final cost centre)*			
Capital items	14,541	11,019	10,913
Wages	26,948	37,675	26,197
Medication and supplies	45,753	43,757	25,195
Subtotal (A)	87,066 (24%)	92,269 (28%)	63,024 (9%)
*Cost of intermediate cost centres allocated to surgery*			
Laboratory	3933	2453	5134
Radiology department	1639	2486	6449
Wards (surgical inpatient services)	80,149	98,775	457,081
Subtotal (B)	85,721 (24%)	103,714 (31%)	468,665 (64%)
*Cost of ancillary departments allocated to surgery*			
Laundry	864	2571	5625
Housekeeping, stores and security	4974	4147	3764
Maintenance and repairs	1152	2208	2751
Utillities	7165	6417	21,709
Pharmacy (excl drugs and supplies)	1277	1294	1088
Kitchen for staff catering	5694	5329	20,918
Transport	150,888	104,493	111,551
Management and administration	2402	1972	2711
Subtotal (C)	194,415 (52%)	133,454 (41%)	204,295 (28%)
Total cost of surgery (A + B+C)	363,202 (100%)	329,437 (100%)	735,985 (100%)

The total cost of surgery at MaDH is similar to that at MuDH ($ 363,000 and $ 329,000, respectively, for the whole year), but the distributions are different due to different costs for the two largest cost centres: surgical inpatient services and transport. MuDH has more senior staff on the wards and at the OT, which results in higher salary costs. MaDH has more vehicles, which largely explains the higher cost of transport.

At $ 736,000, the total cost of surgery at NsDH is about twice as high as at the other two hospitals; this is mainly due to the much higher cost of surgical inpatient services, and in spite of a much lower inpatient load (as shown in Table 2), and a slightly lower total bed capacity (Table 1). The longer duration of stay in combination with a low bed occupancy rate (17%) makes inpatient services at NsDH relatively expensive. The cost of ancillary services at NsDH is also comparatively high, while the direct costs of the operating theatre are somewhat lower than at MaDH and MuDH, mainly due to lower expenditure on CO wages and on medication and supplies.

The annual cost of surgery represents 16–17% of the total cost of the entire hospital for MaDH and MuDH (not shown in the table). At NsDH though, 47% of the total cost of the hospital goes towards surgery or rather to inputs that are supposed to be used for surgery.

The unit cost of the three most common surgical procedures varies considerably amongst the three hospitals, as shown in Table 4. Evacuation from uterus is cheaper than a hernia operation mainly because the former procedure normally does not require the patient to stay at the hospital for more than a day, while hernia cases remain in hospital for a couple of days (3.0 days on average at MaDH, 2.4 days at MuDH, 5.6 days at NsDH). C-sections are more expensive than hernia operations for the same reason: the higher unit cost is partly due to the longer duration of stay (6.3 days on average for a C-section at NsDH, 5.6 days at MaDH, 5.9 days at MuDH). The unit costs of surgical procedures at NsDH are consistently higher than at the other two hospitals. This is because of the higher total cost which has to be shared amongst fewer patients.

**Table 4:** Unit cost of the three most common surgical procedures (in US$)

	Ma DH	Mu DH	Ns DH
Caesarean section	164	251	638
Evacuation from uterus	57	72	143
Hernia procedure	137	164	598

## Discussion

This paper has quantified the monetary cost of providing surgery at district hospitals in Malawi. Much of this cost is hidden to the general public and to health providers themselves, but identifying the various types of resources used in surgery, the quantities involved and their price tags, and allocating the cost of resources that are shared between the operating theatre and other departments to the appropriate services does enable a better understanding of the main cost drivers of surgery.

### Cost of surgery

The total cost of providing district hospital-level surgery in Malawi in the 2013/14 fiscal year ranged from approximately $ 335,000 to almost twice that amount. The cost of running an OT constituted between 10 and 26% of the cost of running the entire hospital. The two main cost drivers of surgery are transport and inpatient services (including catering). This is different from hospitals in most high-income countries, where staff salaries are much higher and which use more advanced surgical techniques that involve expensive equipment and materials. In Malawi, in particular, salaries of civil servants are low: the monthly salary of a clinical officer in 2014/15 varied between MWK 115,000 and 150,000, the equivalent of USD 270–350 at the exchange rate at that time (MWK 428 to USD 1).

The large variation in the distribution of costs over different cost centres observed in this study has also been found in other studies [8–11]. The unit cost of a caesarean section in the three hospitals ranged from $ 164 to $ 638, which is higher than the $ 133 reported by Alkire et al. for Malawi in 2008 [12]. Part of this difference might be due to differences in the methodologies that were used, such as allocation of staff time, allocation of transport costs and depreciation of capital costs. The cost of an evacuation from uterus was between $ 57 and $ 143, which is lower than the $ 239 per patient who received post-abortion care reported by a study in Uganda [13]. The cost of a hernia repair was between $ 137 and $ 598, which is much higher than the $ 59 found in a study in Uganda [14] but comparable to the $ 275 reported for Ghana [15]. Differences in the cost of inputs for instance salary levels of the staff that performs the surgery (medical offers in Ghana and Uganda; clinical officers in Malawi) make cross-country comparisons difficult. But some of the variation may be explained by differences in methodology: other costing studies allocated cost in a ‘top-down approach’, which, in comparison with the present study, also involves more assumptions, hence greater uncertainty.

The model that was built and which takes into account the capital and recurrent cost of all the inputs used for the current levels of output (surgery performed, inpatient care provided) allowed for estimates of the cost implications of scaling up surgery at these hospitals—which will be reported in another paper (forthcoming). It is important to note that all three hospitals appeared to have some surplus capacity to realise a certain scale-up of surgery. This surplus capacity existed both at the operating theatres, since they are not open during all weekdays except for emergencies, and the hospital wards, as indicated by the low bed occupancy rates. The case of Nsanje DH is noteworthy: the high cost of surgery in combination with the relatively small number of surgical cases suggests a considerably less efficient surgical care model compared with the other two hospitals. However, the demand for surgery at Nsanje DH appears less predictable in view of its higher proportion of injury-related and emergency and disability-preventive surgery. This may cause peaks in the demand for surgery and for hospital beds, which this study did not seek to explore.

It is also worth noting that a possible scale-up of surgery requires more than just deploying additional surgical staff or allocating a larger budget to the hospital, even if it was ring-fenced for surgery. For instance, the presence of other OT staff such as anaesthetists is also crucial. It is equally important to have a reliable supply chain management in place and autoclaves in good working conditions so as to ensure timely sterilisation of surgical equipment. These functions are not automatically fulfilled in the event of an increase in the budget dedicated to surgery, as has also been demonstrated in studies elsewhere [16, 17]. A qualitative study amongst medical licentiates who deliver surgery in Zambia, also conducted as part of COST-Africa (another paper by Gajewski et al., forthcoming), showed that task shifting of surgery may be a sustainable response to the unmet surgical needs of rural populations, but it needs to go together with professional recognition, suitable employment conditions and career paths and opportunities of the cadres involved.

Broadening the range and scaling up the volume of major surgery conducted at district hospitals would most likely lead to fewer surgical referrals from district to central hospitals. Apart from the positive health effects this might have [Gajevski et al., forthcoming], it would also imply certain savings, especially on ambulance transport for the evacuation of emergency cases; and less expenditure on the side of central hospitals, where fewer cases of surgery might be expected.

While surgery has for a long time been perceived as complex and expensive, there is now good evidence that many essential surgical interventions can be provided in a cost-effective manner in resource-poor countries [1, 3, 14–18]. Quantification of the monetary cost of providing surgery is essential in the pursuit of expansion and scale-up of surgery in countries where the needs for lifesaving and disability-preventive surgery are unmet. It responds to the call from the Lancet Commission on Global Surgery for LMIC-specific research to investigate how domestic or external funding of surgical care can be used to improve efficiency and performance, and achieve economies of scope and scale with optimum returns on investment. For a typical district hospital in Malawi, we can now calculate the extra budget requirements, and this information may be used at the national level, along with organisational, ethical and political arguments to scale up surgery, so as to eventually meet clinical demand and improve health and welfare. The Lancet Commission has argued that, rather than looking at the extra budget requirements for scaling up surgery as a cost, one should view them as an investment, because of the associated economic gains [3].

This paper has not considered the costs that are often borne by patients to access surgical care, and which for some patients may be catastrophic. To elicit such data, a survey was conducted as part of the COST-Africa project, amongst patients who had undergone surgery at district hospitals and one of the central hospitals in Malawi. The results will be reported separately [Bijlmakers et al., forthcoming], demonstrating that increased access to surgery for rural populations has economic benefits for the patients involved and their families.

### Strengths

This study resulted in a model that allows an automated calculation of the cost implications of different resource inputs of changes in prices and of different scenarios for scaling the delivery of surgery. This makes it a powerful tool. It also enables the calculation of possible savings that can be made if certain resources were used differently (for instance by reducing ambulance trips), and how much extra budget would be required to increase the number of surgical procedures that are undertaken. The model further makes it relatively easy to replicate the study and conduct similar costing studies in other hospitals.

### Limitations

Heterogeneity, uncertainty regarding the robustness of some of the estimates and some model uncertainty related to the criteria used for allocating costs are the main limitations of the study. This is not uncommon in this kind of studies [19]. For example, for the allocation of staff time to surgery, which was based on interviews, uncertainties remained about staff not always being on-site and available, which may also have affected the continuity and quality of services. The absence of hospital staff may happen for various reasons, including workshop attendance, family matters and moon lighting, but reports are often anecdotal [20]. Heterogeneity in the use of drugs and other medical supplies in surgery and in the use of transport cost may also be worth exploring further.

### Policy implications

This study has provided insights into the economic efficiency of health care facilities and had laid a basis to calculate the financial requirements for a possible scaling up of district-level surgery. Hospital managers and national health policy makers may want to take the economies of scale into account and weigh the additional cost of scaling up surgery at district hospitals against the health benefits as well as the savings that would be made. The low bed occupancy rates suggest overcapacity and a potential to reduce the number of beds, reorganise wards and/or reallocate staff, or depending on the catchment population and alternative service providers that may be available, it may point to avoidance of treatment seeking by the population concerned. Staff deployment would need to be seen in relation to the seasonality of certain pathologies and in people’s treatment seeking behaviour [21, 22]. Extension of patients’ duration of stay at the hospital beyond what is required from a medical perspective, as might have been the case in one of the hospitals involved in this study, would need to be discouraged so as to avoid the extra expense.

Further research on the cost of scaling up surgery may inform policy choices, especially if such studies consider the possible changes in patient flows and referral patterns when the range of surgical procedures performed at rural district hospitals is expanded.

## Electronic supplementary material

Below is the link to the electronic supplementary material.

Supplementary material 1 (DOCX 41 kb)

## Footnotes

1 Other recurrent transactions: a term used for the Government budget allocation that hospitals and districts use to cover various types of recurrent expenditure.

## References

[1] Alkire BC, Raykar NP, Shrime MG (2015). Global access to surgical care: a modelling study. Lancet Glob Health.

[2] Shrime MG, Bickler WS, Alkire BC (2015). Global burden of surgical disease: an estimation from the provider perspective. Lancet Glob Health.

[3] Meara JG, Leather AJM, Hagander L (2015). Global Surgery 2030: evidence and solutions for achieving health, welfare and economic development. Lancet.

[4] van Amelsfoort J, van Leeuwen P, Jiskoot P (2010). Surgery in Malawi—the training of clinical officers. Trop Doctor.

[5] Chilopora G, Pereira C, Kamwendo F (2007). Postoperative outcome of caesarean sections and other emergency obstetric surgery by clinical officers and medical officers in Malawi. Hum Resour Health.

[6] Shepard DS, Hodgkin D, Anthony YE (2000). Analysis of hospital costs: a manual for managers.

[7] Conteh L, Walker D (2004). Cost and unit cost calculations using step-down accounting. Health Policy Plan.

[8] Evans CT, Lenhart P, Lin D (2014). A cost analysis of pediatric cataract surgery at two child eye health tertiary facilities in Africa. J AAPOS.

[9] Saronga HP, Duysburgh E, Massawa S (2014). Efficiency of antenatal care and childbirth services in selected primary health care facilities in rural Tanzania: a cross-sectional study. BMC Health Serv Res.

[10] Dalaba MA, Akweongo P, Savadogo G (2013). Cost of maternal health services in selected primary care centres in Ghana: a step down allocation approach. BMC Health Serv Res.

[11] Petrou S, Gray A (2011). Economic evaluation using decision analytical modelling: design, conduct, analysis, and reporting. BMJ.

[12] Alkire BC, Vincent J, Turlington Burns C (2012). Obstructed labor and caesarean delivery: the cost and benefit of surgical intervention. PLoS One.

[13] Vlassoff M, Mugisha F, Sundaram A (2014). The health system cost of post-abortion care in Uganda. Health Policy Plan.

[14] Löfgren J, Mulowooza J, Nordin P (2015). Cost of surgery in a low-income setting in eastern Uganda. Surgery.

[15] Shillcutt SD, Clarke MG, Kingsnorth AN (2010). Cost-effectiveness of groin hernia surgery in the Western Region of Ghana. Arch Surg.

[16] Chao TE, Sharma K, Mandigo M (2014). Cost-effectiveness of surgery and its policy implications for global health: a systematic review and analysis. Lancet Glob Health.

[17] Gallaher JR, Mjuweni S, Cairns B (2015). Burn care delivery in a sub-Saharan African unit: a cost analysis study. Int J Surg.

[18] Grimes CE, Ang-Henry J, Maraka J (2014). Cost-effectiveness of surgery in low- and middle-income countries: a systematic review. World J Surg.

[19] Shrime MG, Alkire BC, Grimes C (2017). Cost-effectiveness in global surgery: pearls, pitfalls, and a checklist. World J Surg.

[20] Cumbi A, Pereira C, Malalane R (2007). Major surgery delegation to mid-level health practitioners in Mozambique: health professionals’ perceptions. Hum Resour Health.

[21] Hlimi T (2015). Association of anemia, pre-eclampsia and eclampsia with seasonality: a realist systematic review. Health Place.

[22] Ewing VL, Lalloo D, Phiri K (2011). Seasonal and geographic differences in treatment-seeking and household cost of febrile illness among children in Malawi. Malaria J.

