# Supplementary material for: The Cost of Providing District-Level Surgery in Malawi
Source: World J Surg. 2017 Aug 8;42(1):46–53. doi: 10.1007/s00268-017-4166-5 (PMC5740194; doi:10.1007/s00268-017-4166-5)
Supplement: Supplementary file 1 — Supplementary material 1 (DOCX 41 kb) [file 268_2017_4166_MOESM1_ESM.docx]

**Online resource 1: Methods**

Supplementary material to article: The cost of providing and scaling up district-level surgery in Malawi

World Journal of Surgery

D. Cornelissen, G. Mwapasa, J. Gajewski, T. McCauley, E. Borgstein, R. Brugha, L. Bijlmakers

Dennis Cornelissen, Radboud University Medical Centre Netherlands, Geert Grooteplein Zuid 10, 6525 GA Nijmegen, The Netherlands

Gerald Mwapasa, University of Malawi, College of Medicine, Mahatma Gandhi, Blantyre, Malawi

Jakub Gajewski, Royal College of Surgeons in Ireland, 123 St Stephens Green, Dublin 2, Ireland

Tracey McCauley, Royal College of Surgeons in Ireland, 123 St Stephens Green, Dublin 2, Ireland Eric Borgstein, University of Malawi, College of Medicine, Mahatma Gandhi, Blantyre, Malawi

Ruairi Brugha, Royal College of Surgeons in Ireland, 123 St Stephens Green, Dublin 2, Ireland

*Leon Bijlmakers, Radboud University Medical Centre Netherlands, Geert Grooteplein Zuid 10, 6525 GA Nijmegen, The Netherlands (Leon.Bijlmakers@radboudumc.nl)

**Corresponding author*

**Materials and methods**

*Setting and scope*

The three hospitals involved in this study were visited between November 2014 and March 2015 by one of the researchers (DC) to conduct interviews and collect data from existing hospital registers and financial records. All presented data pertain to the period 1 July 2013 to 30 June 2014, which corresponds with one entire financial year. Conversion of local prices to US dollars was based on the exchange rate on 1^st^ January 2014, which was 428 Malawian Kwachas to one US dollar.

*Costing method*

For each of the three hospitals the step-down method was used for costing [6,7]. It involves six steps: 1) define the final product(s) of interest; 2) define the final, intermediate and ancillary cost centres; 3) identify all inputs and calculate the full costs of each input; 4) assign inputs to the appropriate cost centres; 5) allocate all direct costs and appropriate proportions of the costs of the inputs used at ancillary and intermediate cost centres to the final cost centre; and 6) calculate the unit cost for each service provided at the final cost centre. The following sections describe how we implemented each of these steps. This is then followed by a brief description of how we used the data set to calculate the costs of various scenarios for scaling up surgery.

*Step 1: Defining the final products*

We defined the final products as surgical procedures and associated post-operative care. Four main categories of surgical procedures were distinguished: pregnancy-related & obstetric surgery, emergency & disability-preventive surgery, injury-related surgery and other forms of surgery. Each of these categories contains several surgical procedures, which are listed in Box 1 in the main article. Not all surgical procedures require post-operative care. Patients who underwent a minor procedure were assumed to have stayed at the hospital for one night. Patients who received minor surgical treatment at the outpatient department (at MuDH and NsDH) were excluded. Theatre registers were used to obtain the total number of patients who underwent surgery at the theatre complex during the 12-months period, by type of procedure. Monthly reports from the health management information system were used to obtain the number of hospital admissions, patients’ length of stay and the number of outpatient visits. Ward registers served to obtain the length of stay of surgical patients by type of procedure.

*Step 2: Defining final, intermediate and ancillary cost centres*

All hospital departments were considered cost centres. The operating theatre was defined as the final cost centre. Intermediate cost centres are departments that render services indirectly, but not exclusively to patients served at the final cost centre (in our case surgical patients at the operating theatre). These include the laboratory department and radiology, as well as the inpatient wards, since none of the three hospitals have designated surgical wards. Part of the costs of these intermediate cost centres needed to be allocated to the operating theatre, with the remainder going to other hospital departments.

Box 2: Types of expenditure in operating theatre, intermediate cost centres and ancillary departments, with criteria used for allocating costs to surgery

| **Final cost centre** | **Cost allocation criteria** |
| --- | --- |
| Operating theatre | Capital items and medication fully allocated to surgery;  Wages of surgical staff allocated in relation to actual time spent in theatre |
| **Intermediate cost centres** | **Criteria to allocate costs to final cost centre** |
| Laboratory | Number of investigations done for surgical patients divided by the total number of investigations |
| Radiology | Number of investigations done for surgical patients divided by the total number of investigations |
| Inpatient wards (including patient meals from kitchen department) | Inpatient days of surgical patients divided by the total number of inpatient days  Patient catering based on distribution surgical : non-surgical inpatient days |
| **Ancillary cost centres** | **Criteria to divide costs between final and intermediate cost centres** |
| Laundry | Surgical inpatient days as a percentage of total inpatient days |
| Housekeeping & security;  Maintenance & repairs | Allocation based on surface area of departments (in square metres) proportional to the total surface area of hospital |
| Utilities (electricity, water) | Allocation proportional to the weighted total surface area of hospital, with departments that are open day and night being given 50% more weight than departments that close at night. |
| Pharmacy (excluding drugs and supplies) | Number of surgical patients divided by total number of patients |
| Kitchen for staff catering | Allocation based on weighted patient days ^a^ |
| Transport | 50% allocated to hospital (remaining 50% to district health management); further divided between final cost centre and intermediate cost centres based on weighted patient days ^a^ |
| Management & administration | 50% allocated to hospital (remaining 50% to district health management), further divided between final cost centre and intermediate cost centres based on weighted patients days ^a^ |

^a^  Each surgical procedure and each inpatient day (surgical or non-surgical) was weighted as 1. An outpatient visit was weighted as 0.25.

Departments that serve both the final cost centre and intermediate cost centres were considered ancillary cost centres; examples of these are laundry and administration. The administration cost centre was divided into several sub-centres: housekeeping & security, utilities (electricity, water, telephone) and hospital & district health management. The first column in Box 2 presents the full list of final, intermediate and ancillary cost centres.

*Step 3: Identifying and costing all inputs*

The inputs used at the various cost centres of the hospital were divided into capital costs, monthly salaries and supplies. Box 3 lists these inputs and indicates the sources used to obtain data about their costs.

Box 3: Sources of input data

| **Input parameter** | **Source of cost data** |
| --- | --- |
| Volume of surgical procedures, per type | Operating theatre register |
| Length of stay, per type of surgical procedure | Ward registers |
| Volume of admissions, inpatient days and OPD visits | Records provided by health management information system officer |
| Hospital staff composition and wages  Physical infrastructure and inventory (capital items)  Consumption of medical and non-medical supplies  Service output of various ancillary departments & support services  District level services: transport and management activities | District health office: registers and interviews (notably the district health officer, the district medical officer and the district nursing officer), personal emolument documents |
| Replacement costs of capital items | Price lists from MoH Central Medical Stores, UNICEF ^a^ and WHO ^b^ |
| Surface area of various hospital departments | Tape measurements |

^a^ UNICEF Supply Catalogue. Available at <https://supply.unicef.org/unicef_b2c/app/>

^b^ WHO, 2011. Core medical equipment. <http://www.who.int/medical_devices/en/index.html>

For costing the inputs several assumptions were made. Firstly, for buildings a standard capital cost of $100 per square metre surface area was used. Secondly, since salaries and allowances varied quite substantially per category of staff (because of differences in seniority), we used the average monthly wages and average allowances of each category of staff (clinical officers, anaesthetists, nursing officers, attendants). Thirdly, staff interviews were held to obtain estimates of the typical amounts of pharmaceuticals and other supplies used for various types of surgical procedures and post-operative. We explicitly asked theatre staff to take wastage and episodes of stock-outs into account. An example of the latter is that at two hospitals clinical officers reported the use of chrome sutures in about half of all the procedures that involved caesarean section combined with bilateral tubal ligation. For the other half of these procedures they used vicryl sutures as a less expensive alternative or in case they ran out of chrome sutures. Fourthly, several medical equipment items had surpassed their expected useful life time but were still functional. Estimates of the cost of replacing these items were sourced and used, even though this yielded hypothetical figures since replacement may not happen in reality: hospitals in Malawi tend to rely on donations as the Ministry of Health has limited budgetary space to purchase new equipment. Finally, several assumptions were made regarding the annualisation of capital costs. It was assumed that buildings had 30 non-transferrable useful years of life; furniture 10 years; vehicles and large items of equipment, such as anaesthesia and diathermy machines, five years; and small items of equipment one year. In order to take depreciation of capital inputs into account we assumed a discount rate of 6%. Using a standard table^[[1]](#footnote-1)^, we derived the following annualisation factors: buildings 14%, furniture 7%, large equipment 4% and small equipment 1%.

*Step 4: Assigning inputs to cost centres*

Capital costs for buildings were allocated to cost centres based on the surface areas of the respective departments. Maintenance of buildings was a separate cost centre, with cost allocation based on surface area (square metres, measured manually with a tape measure). The cost of maintenance of equipment was allocated to the departments that actually used the equipment. Vehicle maintenance was charged to the transport department.

The salaries and allowances of theatre nurses, anaesthetists, cleaners and porters who work in the operating theatre only were fully assigned to the theatre department. Since they have no other duties, the cost of idle time of operating theatre staff was also allocated fully to theatre. Clinical officers have duties in other departments as well, for example in the outpatient department, in the wards. Their wages were allocated in proportion to the time they worked in the operating theatre or the wards for post-surgical care. This required estimates of the time clinical officers spent on surgery and these were obtained through interviews with three staff members in each of the hospitals (one clinical officer, one theatre nurse and one anaesthetist). One-hundred percent of the costs of capital items and supplies used in the operating theatre was assigned to the final cost centre.

*Step 5: Stepwise cost allocation*

The costs of the various ancillary cost centres were divided between the relevant intermediate cost centres and the final cost centre, on the basis of the criteria that are presented in Box 3 (second column). Some of these criteria were straightforward and do not need further explanation, such as the allocation of costs of the diagnostics department proportionally to the percentage of investigations that were done for surgical patients. For other criteria, however, we had to make assumptions that warrant further explanation.

The costs for patient catering and laundry services were divided between the operating theatre and the inpatient wards based on the relative number of patient days. Patient days on which a surgical procedure occurred were given twice as much weight as a regular inpatient day, and each out-patient was given a quarter of the weight of one regular inpatient day. These assumptions were based on observations of the relatively high workload to attend to surgical patients compared to non-surgical inpatients and out-patients, respectively. The kitchen departments provide meals and teas to inpatients as well as staff. Mangochi DH procures staff meals from a local restaurant. ­­­­­­The costs of utilities (water and electricity) were shared among all departments based on their relative surface area, whereby the wards that are open day and night received a higher weight of 1.5, with lower weights of 1.0 for all departments that are open during the day only.

It proved challenging to find an appropriate criterion which would determine how much of the total cost of ambulance services and other types of transport (calculated from expenditure for fuel & lubricants, maintenance and drivers’ salaries) would need to be allocated to surgery. This was because vehicle logbooks showed that trips often had multiple purposes; for instance ambulance trips for the evacuation of patients may be combined with transportation of supplies or supervision visits of nearby health centres. Sometimes the purpose of trips was not clear from the logbooks. For patients who were referred to undergo elective surgery at another hospital it was not uncommon that two or more patients were transported in a single ambulance trip. In case of a surgical emergency staff members who were on night or weekend call were sometimes picked up from their homes by a hospital vehicle. Based on interviews with members of the respective district health management teams, half of the transportation cost was allocated to the hospital while the other half was allocated to health facilities elsewhere in the district which do not have their own vehicles. As with the catering and laundry services, the hospital’s transport costs were then divided between surgery and other inpatient departments based on weighted patient days to reflect the relative workloads. Lastly, half of the management costs was allocated to the district hospital, with the other half shared among all other health facilities in the district.

*Step 6: Calculating the total cost of surgery and unit costs of specific surgical procedures*

The total cost of surgery, comprising the cost of the procedures themselves plus the cost of surgical inpatient care, was calculated by adding up the direct cost and all cost allocations from ancillary and intermediate cost centres. This total cost was then divided between all the surgical procedures performed during the year, taking into account the average duration of the various types of procedures, as reported in theatre staff interviews.

As an illustration of this method, consider a theatre of which the total cost for the year was $30,000 and assume it was open during the year for a total of 1000 hours. If 200 hernia operations were performed, each taking one hour on average, the unit cost for a hernia operation would be

$$\frac{\$30,000\cdot\frac{200}{1000}}{200}=\$30$$

*Electronic model, with data organised in linked spreadsheets*

An electronic model was constructed in MS Excel with several linked spreadsheets that contained the cost centres, the volumes of inputs used, unit costs and service production (number of surgical procedures performed by type, number of admissions and length of stay of patients). The allocation criteria described in step 5 above were translated into automatic formulae, which resulted in the total cost of surgery and unit costs per type of surgical procedure, including the cost of associated post-surgical care.

*Forecasting the cost of scaling up surgery*

We were interested in the cost implications of two different scenarios for scaling up surgery at the three hospitals: 1) increasing the number of surgical procedures that are performed by 20% or 50%; and 2) expanding the surgical capacity by deploying two extra surgically trained clinical officers. Since the spreadsheets in the model were linked and all calculations automated, any change in the parameters for the two scenarios of interest resulted automatically in new cost estimates and the increments compared to the current scenario.

1. Creese A. and Parker D (eds; 1994): Cost analysis in primary health care – a training manual for programme managers. WHO, Geneva. [↑](#footnote-ref-1)
